# Self-Reevaluation and Anticipated Regret Did Not Change Attitude, Nor Perceived Distance in an Online Context

**DOI:** 10.3389/fpsyg.2016.02038

**Published:** 2017-01-11

**Authors:** Rik Crutzen, Dianne Cyr, Sarah E. Taylor, Eric Lim, Robert A. C. Ruiter

**Affiliations:** ^1^Department of Health Promotion, Maastricht University/CAPHRIMaastricht, Netherlands; ^2^Beedie School of Business, Simon Fraser UniversityVancouver, BC, Canada; ^3^Department of Health Promotion, Maastricht University/CAPHRIMaastricht, Netherlands; ^4^UNSW Australia Business School, University of New South WalesSydney, NSW, Australia; ^5^Department of Work and Social Psychology, Faculty of Psychology and Neuroscience, Maastricht UniversityMaastricht, Netherlands

**Keywords:** behavior change methods, self-reevaluation, anticipated regret, self-reference, eHealth

## Abstract

Internet-delivered interventions can be effective in changing behavior, but more research is needed on effective elements of behavior change interventions. Moreover, although anonymity is one of the advantages of using an online context, it might also increase the perceived distance between the participant and the intervention. Hence, the current study investigated whether the behavior change methods of self-reevaluation and anticipated regret can be used to narrow the perceived distance and, ultimately, foster attitude change. A 3 × 3 factorial between-persons design with an additional control group was used (*N* = 466), resulting in a total of 10 conditions (n's ranging from 43 to 49). The first factor manipulated is assessment of self-image; cognitive, affective, or the combination of both. The second factor manipulated is behavioral focus; self-image with behavior, without behavior or both with and without behavior. Post-test measurements were conducted immediately after the manipulation. The key finding of the current study is that the behavior change methods of self-reevaluation and anticipated regret did not have an impact on changes in attitude toward oral contraceptive use, nor on the distance perceived by participants. Despite the null results, the current study contributes to the body of evidence regarding self-reevaluation and anticipated regret, which can be integrated in meta-regressions of experimental studies to advance behavior change theory.

## Introduction

Persuasive information systems are designed to alter, reinforce, or shape users' attitudes and/or behaviors, without resorting to coercion or deception (Oinas-Kukkonen and Harjumaa, 2009). Within the field of health psychology, such systems can be operationalized as Internet-delivered interventions aimed at promoting healthy behavior. These interventions can be effective in changing behavior, but a systematic review of reviews concluded that more research is needed on effective elements of behavior change interventions (Kohl et al., 2013). In other words, effectiveness of interventions cannot yet be unambiguously attributed to isolated elements, also known as active ingredients of an intervention (Peters et al., 2015). Furthermore, it remains to explored what elements work in what situation (e.g., different contexts) and for whom (e.g., different target populations) (Kohl et al., 2013).

Effective elements of an intervention (i.e., the active ingredients) are typically designed using evidence-based behavior change methods, which are general techniques of change that can be applied across different contexts and populations. For example, persuasive communication is all around us targeting different populations in all kinds of settings (e.g., health education, commercial advertisements, parent-child communication). These methods are not directly aimed at behavior, but target the underlying causes (or determinants) of behavior that are reflected in our thoughts and feelings (e.g., perceived pros and cons of the behavior). Practical applications are the translations of theoretical methods into practical intervention elements that fit the intervention context and target population characteristics. For example, the use of persuasive communication [theoretical method] can be translated in a role play presented through a video clip on the Internet [practical application], which would fit an intervention context where computers are widely available. But that practical translation would not be optimal when the health intervention is targeting an at risk population in rural areas in countries where access to Internet and computers is less common. Importantly, a practical application must comply with the theoretical method's parameters for use. When these parameters are not properly translated from theoretical method into practical application, the effectiveness of a method is undermined and may even be counter productive (Bartholomew Eldredge et al., 2016). For example, the effectiveness of fear arousal [theoretical method] depends on the perceived threat and whether people feel capable of performing the recommended action to avert the threat (i.e., perceived efficacy) [parameters for use]. If the condition of efficacy is not met, the use of fear arousal may result in feelings of hopelessness, which in turn results in defensive responses such as risk denial and message avoidance (Peters et al., 2013). Effectiveness of persuasive communication [theoretical method] depends on, for example, messages being relevant and not too discrepant from the beliefs of the individual [parameters for use].

The current study focuses on narrowing the perceived distance in order to foster attitude change in an online context. Anonymity is one of the advantages of using an online context, because it reduces the threshold to use Internet-delivered interventions (Kohl et al., 2013). However, it might also increase the perceived distance between the participant and the intervention. When interacting with a social partner who does not respond in a reciprocal manner, a distance is perceived between the participant and the social partner (Trope and Liberman, 2010). The idea of distance having an impact on attitude originated from previous work looking at the relationship between physical distance and attitude. For example, a smaller physical distance when communicating with someone, resulted in a more positive attitude toward that person (Mehrabian, 1968). Later on, these finding were confirmed for perceived distance. For example, participant were trained to pull a joystick toward themselves or to push it away from themselves when presented with photographs of people from different ethnic backgrounds. Pulling a joystick toward themselves, and thus reducing the perceived distance, resulted in a more positive attitude (i.e., a reduction in implicit prejudice), which also resulted in increased immediacy when interacting with people from different ethnic backgrounds afterwards (Kawakami et al., 2007). It has been suggested that people interact with computer interfaces, such as in an online context, as though they were social partners (Nass and Moon, 2000). Therefore, when participating in an Internet-delivered intervention and interacting with the computer as a social partner, it is reasonable to believe that a distance will be perceived by the user between him or her and the communicator, because the computer cannot respond in the socially expected manner. This perceived distance may cause an individual to consciously or unconsciously reject advice (Foster and Ford, 2003). In other words, the greater the perceived distance between a participant and an intervention, the less likely it is that an intervention is able to exert influence over a user's attitude (Al-Natour et al., 2011).

We propose self-reevaluation as a method to narrow the perceived distance in an online context in order to foster attitude change. Self-reevaluation involves reappraisal of how engaging in, or refraining from, a certain behavior is part of one's identity (Longmire-Avital et al., 2010). The potential working mechanisms of self-reevaluation can be derived from previous work: more *self-reference* resulting from framing objects or events in an egocentric manner (Rogers et al., 1977) is one of the experiential processes of change (Prochaska et al., 2008) and less *psychological distance* because framing objects or events in an egocentric manner reduces the perceived distance between the object or event and the self (Liberman et al., 2007). Self-reevaluation concerns encouraging cognitive *and* affective assessments of one's self-image with *and* without certain behavior. It leads to answering questions such as how would I feel behaving this way? What would I think about myself if I would not behave this way (Prochaska et al., 2008)?

A closely related method is fostering anticipated regret, which stimulates people to focus on their feelings (i.e., affective assessment) after unintended risky behavior (i.e., with a certain behavior), before any losses actually materialize (Richard et al., 1996). For example, a sex educator might ask people to image how they would feel after having had unsafe sex (Bartholomew Eldredge et al., 2016). This differs from self-reevaluation as its parameters for use concern *only* affective assessment (not cognitive) of one's self-image with an unhealthy behavior (not without). In other words, anticipated regret overlaps with self-reevaluation in terms of affective assessment of self-image but does not require cognitive assessment. In terms of behavioral focus, anticipated regret only requires self-image with a certain behavior, whereas self-reevaluation also requires self-image without a certain behavior. Both self-reevaluation and anticipated regret can be used to change attitudes (Kok et al., 2016). It is unclear, however, whether the requirements of self-reevaluation have added value to anticipated regret, both in terms of perceived distance and, ultimately, attitude change (irrespective of whether this change is positive or negative). Hence, we use a full-factorial design to explore the optimal combination of parameters for use in an online context (Peters et al., 2015) and to investigate the impact of these methods on attitude change. This contributes to the evidence base on whether and how these methods can be used in an online context.

## Preparatory study

In order to investigate the extent to which there is an impact on attitude change, a target behavior is chosen in which there is a certain amount of attitudinal ambivalence. This is because attitudes are more malleable when they are ambiguous or ambivalent (Petty and Cacioppo, 1986). Therefore, the focus is on young women's attitudes toward oral contraceptive pills (OCP). Several beliefs are known to influence the attitudes of women toward OCP, such as beliefs regarding the accessibility and effectiveness of OCP (Landau et al., 2006) and the side effects and risks associated with OCP use (Murphy et al., 1995; Lee and Jezewski, 2007). One person can, for example, be convinced about the effectiveness of OCP, but at the same time worry about possible side effects. This might lead to attitudinal ambivalence.

A preparatory study was conducted before the main study. The aim of this preparatory study was twofold. First, to conduct a belief elicitation procedure (as recommended by Fishbein and Ajzen, 2010) to ensure that the previously developed scale to measure attitude toward OCP (Herold and Goodwin, 1980) includes all relevant beliefs. Second, to assess scale quality of measurements before using them in the main study and to explore the relations between these measurements.

### Methods

A small-scale cross-sectional survey was conducted.

#### Participants

Participants were recruited through an online panel (i.e., Prolific Academic). A total of 50 women between 18 and 25 years were eligible to participate. After giving informed consent, participants completed the measurements described below. Participants received an incentive (i.e., £2) upon completion of the preparatory study. Data from two participants were removed from the analyses due to unsatisfactory completion (i.e., > 50% missing data).

#### Measurements

The survey consisted of the measurements described below.

##### Belief elicitation

The belief elicitation procedure was conducted by means of the three questions suggested by Fishbein and Ajzen (2010): “What do you see as the advantages of using oral contraceptive pills?”; “What do you see as the disadvantages of using oral contraceptive pills?”; and “What else comes to mind when you think about using oral contraceptive pills?” The questions used a free-response format.

##### Attitude

Attitude was assessed by a previously developed scale (Herold and Goodwin, 1980) consisting of 14 items using a semantic differential scaling method (e.g., “I believe oral contraceptive pills are harmful/beneficial”).

##### Self-reference

Self-reference was assessed by four items that had to be answered on a 5-point scale (e.g., “Information you would gather from Wikipedia/Facebook/a personal friend pertaining to oral contraceptive pills is personally relevant to me”; strongly disagree–strongly agree). These items were derived from a previous study (Stanczyk et al., 2013).

##### Psychological distance

Psychological distance was assessed by five items that had to be answered on a 5-point scale (e.g., “Information you would gather from Wikipedia/Facebook/social interaction with a personal friend pertaining to oral contraceptive pills is written by others who have personal traits similar to mine”; strongly disagree–strongly agree). These items were based on the conceptualization of psychological distance in propinquity theory (Newcomb, 1956).

Items regarding self-reference and psychological distance were assessed three times, referring to information on Wikipedia, Facebook, and a personal friend respectively. The idea behind this was to gain more insight into whether there is indeed more self-reference/less psychological distance regarding information gathered when interacting with a personal friend in comparison to information read on the Internet. Wikipedia and Facebook were used as two well-known sources on the Internet. The scores on items regarding self-reference and psychological distance can be interpreted in such a way that higher scores are indicative of lower perceived distance.

#### Analyses

First, to ensure that the previously developed scale to measure attitude toward OCP (Herold and Goodwin, 1980) includes all relevant beliefs for our target group, the answers to the three open-ended belief elicitation questions were compared to the items of the previously developed scale. Second, to assess scale quality of measurements before using them in the main study, we verified dimensionality and subsequently presents McDonald's omega as a less biased alternative to Cronbach's alpha (Crutzen and Peters, 2016). More specifically, omega_hierarchical_ estimates factor saturation based upon the sum of the squared loadings of items on the general factor (McDonald, 1999). Finally, to explore the relations between measurements, we inspected the correlation between self-reference and psychological distance). Moreover, using dependent sample *t*-tests, we assessed whether there is indeed more self-reference/less psychological distance regarding information gathered when interacting with a personal friend in comparison to information read on the Internet.

### Results

First, all answers to the belief elicitation questions were covered by the items in the previously developed scale, except for “control over menstruation” as a reason to use oral contraceptive pills. Hence, an item covering this belief was added to the scale used in the main study. Second, omega_*hierarchical*_ was deemed appropriate for the measurements to be included in the main study: attitude (Ω = 0.77), self-reference (Ω = 0.84; 0.86;0.91), and psychological distance (Ω = 0.76;0.84;0.76). Finally, the association between self-reference and psychological distance was very strong (*r* = 0.70). Dependent samples *t*-tests revealed that there was more self-reference regarding information gathered when interacting with a personal friend (*M* = 4.17, *SD* = 0.84) in comparison with information read on Wikipedia [*M* = 3.35, *SD* = 0.87, *t*_(46)_ = 13.46, *p* < 0.001, *r* = 0.89] or Facebook [*M* = 3.29, *SD* = 0.92, *t*_(46)_ = 8.44, *p* < 0.001, *r* = 0.78]. The same applies to psychological distance when interacting with a personal friend (*M* = 4.20, *SD* = 0.59) in comparison with information read on Wikipedia [*M* = 3.05, *SD* = 0.61, *t*_(47)_ = 7.26, *p* < 0.001, *r* = 0.73] or Facebook [*M* = 3.43, *SD* = 0.77, *t*_(47)_ = 9.49, *p* < 0.001, *r* = 0.88]. All these effects were very large (Rosenthal, 1996). In sum, all measurements were deemed appropriate for use in the main study.

## Main study

We have preregistered the study protocol at https://osf.io/pgv38/ before data collection of the main study. Furthermore, materials used in this study as well as non-identifiable data, syntax, and output of the analyses are available at https://osf.io/s9hu3/. These efforts are taken to acknowledge a recent call for full disclosure to maximize scrutiny, foster accurate replication, and facilitate future data syntheses (e.g., meta-analyses) (Crutzen et al., 2012; Peters et al., 2012). Ethical approval was granted by the Simon Fraser University Research Ethics Board (file number: 2015s037) as well as the Ethical Committee Psychology of Maastricht University (ECP-149).

### Methods

A 3 × 3 factorial between-persons design with an additional control group resulted in a total of 10 conditions. The first factor manipulated is assessment of self-image; cognitive, affective, or the combination of both. The second factor manipulated is behavioral focus; self-image with behavior, without behavior or both with and without behavior. These factors reflect the possible combinations of parameters for use of self-reevaluation. Anticipated regret only requires affective assessment of self-image with behavior. Self-reevaluation is not stimulated at all in the control group.

#### Participants

Participants were recruited through an online panel. Women between 18 and 25 years were eligible to participate. G^*^Power 3.1 was used for an a priori power analysis for *F*-tests (Faul et al., 2007). Based on a small-to-medium effect size (*f* = 0.175; effect size used in analysis of variance), an alpha of 0.05, a power of 0.90, 10 conditions, and three comparisons, a total sample size of 467 is required.

#### Procedure

After giving informed consent, participants completed pre-test measurements regarding their *attitude* toward OCP and *attitudinal ambivalence*. Subsequently, all participants were required to read an information text about OCP, which served as a way of introducing participants to the topic they should reflect on during self-reevaluation. Thereafter, except for participants in the control group who only received this information text, they were asked to comply with various levels of self-reevaluation in line with the factors reflecting the parameters for use. Post-test measurements concern a manipulation check to assess the extent to which the manipulation succeeded in *stimulating self-reevaluation, self-reference, psychological distance, attitude* toward OCP, and *attitudinal ambivalence*. Finally, participants were debriefed. Participants received an incentive (i.e., £3) upon completion of the main study.

#### Manipulations and material

##### Information text

The information text about OCP was based on information from The United States Centre for Disease Control and Prevention, The American College of Obstetricians and Gynecologists and Stanford Hospital and Clinics. The information text contained objective medical evidence regarding the benefits and risks associated with OCP. The information text is available at https://osf.io/ksdjy/.

##### Self-reevaluation

The manipulations to stimulate self-reevaluation were:

[cognitive assessment of self-image with behavior] Please think about yourself if you were ***using*** oral contraceptive pills. How do you imagine you would ***think*** about yourself? What kind of ***thoughts*** would you have?
[cognitive assessment of self-image without behavior] Please think about yourself if you were ***not using*** oral contraceptive pills. How do you imagine you would ***think*** about yourself? What kind of ***thoughts*** would you have?
[affective assessment of self-image with behavior] Please imagine if you were ***using*** oral contraceptive pills. How do you imagine you would ***feel***? What kind of ***emotions*** would you experience?
[affective assessment of self-image without behavior] Please imagine if you were ***not using*** oral contraceptive pills. How do you imagine you would ***feel***? What kind of ***emotions*** would you experience?

#### Measurements

All items included in the measurements are available at https://osf.io/5h8pi/. Most of these items were used in the preparatory study as well.

##### Attitude

Attitude was assessed by a previously developed scale (Herold and Goodwin, 1980). Based on the belief elicitation procedure in the preparatory study, one additional item was added concerning adequate control over menstrual cycle. All 15 items had to be answered on a 7-point scale items using a semantic differential scaling method (e.g., “I believe oral contraceptive pills are harmful/beneficial”).

##### Attitudinal ambivalence

Attitudinal ambivalence was assessed (cf., Thompson et al., 1995), contrasting favourableness regarding positive and negative aspects of OCP. All six items had to be answered on a 4-point scale (e.g., 1 = not at all favorable–4 = extremely favorable).

##### Stimulating self-reevaluation

Two items were used as a manipulation check to assess the extent to which self-reevaluation stimulates the expression of thoughts (as a manipulation check for cognitive assessment) and emotions about OCP (as a manipulation check for affective assessment). These items had to be answered on a 7-point scale (ranging from “strongly disagree” to “strongly agree”).

##### Self-reference and psychological distance

Self-reference is assessed by four items that were answered on a 7-point scale (strongly disagree–strongly agree), and psychological distance by five items. These items were similar to the preparatory study. Items regarding self-reference and psychological distance were assessed a total of three times. In the first instance the items referred to the information provided in this study, and in the subsequent two instances the items referred to information from Facebook and information from a personal friend. This allowed comparison with other delivery channels. The scores on items regarding self-reference and psychological distance can be interpreted in such a way that higher scores are indicative of lower perceived distance.

#### Analyses

Before conducting the analyses, data from participants was excluded if there was >10% missing data for a specific participant. Data from a specific measurement (i.e., attitude, attitudinal ambivalence, self-reference, and psychological distance) was excluded if >2 items per measurement were missing. This was specified in the protocol. To assess scale quality for attitude, self-reference, and psychological distance, we again verified dimensionality and subsequently present omega_hierarchical_, which estimates factor saturation based upon the sum of the squared loadings of items on the general factor (McDonald, 1999), and eigenvalues to estimate the explained variance (Kaiser, 1960). Scale quality—contrary to what the term might seem to suggest—is not a characteristic of a scale as such, but depends on the interpretation of scale scores in a specific study (Crutzen and Peters, 2016). Hence, this needs attention every time a measurement is used. If scale quality was deemed appropriate, the mean scores of the items per measurement were used for further analyses.

As a manipulation check, independent samples *t*-tests were used to assess whether the two items regarding stimulating self-reevaluation scored higher in conditions in which respectively cognitive and/or affective assessment of self-image were manipulated. To compare self-reference and psychological distance between this study and other delivery channels (Facebook and personal friend), we used paired samples *t*-tests.

The primary outcome is absolute attitude change between pre- and post-test measurement. To calculate this outcome, we subtracted the mean of all attitude items at pre-test (ranging from 1–7) from the mean of all attitude items at post-test (ranging from 1–7) and took the absolute value of this difference. *Absolute* attitude change is chosen, because the focus of the study is on fostering attitude change, irrespective of whether participants' attitude is more positive or more negative toward OCP. Using or not using OCP is not valued as being “good” or “bad” behavior. Based on a reviewer's suggestion, we also conducted post-hoc analyses using attitude change divided by the attitude at pre-test as an outcome. Univariate analysis of variance (ANOVA) was used to assess whether there was a difference between conditions. We initially tested a model with both factors (self-image and behavioral focus) as well as the interaction between them. If this interaction was non-significant, we ran the model again without the interaction term. Moreover, we report three primary comparisons, which were a priori specified contrasts: (1) the optimal combination of parameters for use for self-reevaluation vs. the control group; (2) for anticipated regret vs. the control group; and (3) for self-reevaluation vs. anticipated regret. Finally, we also compared the control group to all other conditions. The same analyses were conducted using self-reference and psychological distance as outcome variables, as these were deemed potential working mechanisms for self-reevaluation.

The secondary outcome is attitudinal ambivalence. Using the procedures described to calculate ambivalence scores (Thompson et al., 1995), this variable was dichotomized in participants being ambivalent (1) or not (0). When looking at the change between pre- and post-test measurement, there are three possible outcomes: participants become less ambivalent (−1), do not change (0), or become more ambivalent (1). Nominal regression models were used to assess whether there was a difference between conditions. We used the same strategy as with the primary outcome: we initially tested a model with both factors (self-image and behavioral focus) as well as the interaction between them. If this interaction was non-significant, we ran the model again without the interaction term.

### Results

A total of 515 young women participated in the main study. Data from 49 of these young women (9.5%) were removed from the analyses due to unsatisfactory completion according to the protocol (i.e., >10% missing data). This resulted in inclusion of data from 466 participants (90.5%) in the analyses. The mean age of these women was 21.3 years (SD = 1.77). The scale quality for attitude, self-reference, and psychological distance was deemed appropriate for further analysis (Table 1).

**Table 1:** **Scale quality for attitude, self-reference, and psychological distance**.

**Measurement**	**M (SD)**	**Eigenvalue**	**Ω**	**Correlations**							
				**1**	**2**	**3**	**4**	**5**	**6**	**7**	**8**
1. Attitude (pre-test)	5.48 (1.04)	7.93	0.79	−	0.92	0.43	0.20	0.36	0.32	0.11	0.28
2. Attitude (post-test)	5.56 (1.07)	8.76	0.82		−	0.41	0.20	0.37	0.31	0.12	0.32
3. Self-reference (this study)	5.09 (1.32)	2.68	0.83			−	0.39	0.42	0.49	0.24	0.32
4. Self-reference (Facebook)	3.76 (1.30)	2.78	0.84				−	0.38	0.20	0.68	0.30
5. Self-reference (personal friend)	5.78 (1.17)	3.15	0.89					−	0.22	0.25	0.68
6. Psychological distance (this study)	4.56 (0.97)	3.67	0.85						−	0.21	0.27
7. Psychological distance (Facebook)	3.88 (1.22)	3.95	0.87							−	0.37
8. Psychological distance (personal friend)	5.51 (1.09)	3.96	0.85								−

#### Manipulation check

There is no difference in the extent to which self-reevaluation stimulated the expression of thoughts about OCP [*t*_(416)_ = −0.01, *p* = 0.99, *r* < 0.01] between participants in conditions in which cognitive assessment of self-image was manipulated (*M* = 5.65, *SD* = 1.29) vs. participants in conditions in which this was not manipulated (*M* = 5.65, *SD* = 1.36). There is a difference in the extent to which self-reevaluation stimulated the expression of emotions about OCP [*t*_(414)_ = −10.83, *p* < 0.001, *r* = 0.47]. Participants in conditions in which affective assessment of self-image was manipulated scored higher on stimulating self-reevaluation (*M* = 5.58, *SD* = 1.25) in comparison with participants in conditions in which this was not manipulated (*M* = 3.95, *SD* = 1.79).

Self-reference in this study scored lower in comparison with self-reference regarding information from a personal friend [*t*_(464)_ = −11.01, *p* < 0.001, *r* = 0.46], but higher in comparison with self-reference regarding information from Facebook [*t*_(463)_ = 19.87, *p* < 0.001, *r* = 0.68]. Also psychological distance in this study scored lower in comparison with psychological distance regarding information from a personal friend [*t*_(465)_ = −16.45, *p* < 0.001, *r* = 0.61], but higher in comparison with psychological distance regarding information from Facebook [*t*_(463)_ = 10.47, *p* < 0.001, *r* = 0.44].

#### Primary outcome

The absolute attitude change between pre- and post-test measurement was 0.28 (SD = 0.33). However, there is no interaction between the factors self-image and behavioral focus [*F*_(4, 455)_ = 0.93, *p* = 0.45, $\eta_{p}^{2}$ = 0.008], nor a main effect of self-image [*F*_(2, 459)_ = 1.69, *p* = 0.19, $\eta_{p}^{2}$ = 0.007] or behavioral focus [*F*_(2, 459)_ = 2.59, *p* = 0.08, $\eta_{p}^{2}$ = 0.011]. The contrasts revealed no difference either between (1) the optimal combination of parameters for use for self-reevaluation vs. the control group [*t*_(455)_ = −0.41, *p* = 0.69, *r* = 0.02]; (2) for anticipated regret vs. the control group [*t*_(455)_ = 0.93, *p* = 0.35, *r* = 0.04]; and (3) for self-reevaluation vs. anticipated regret [*t*_(455)_ = −1.32, *p* = 0.19, *r* = 0.06], nor for the comparison between the control group and all other conditions [*t*_(455)_ = 0.14, *p* = 0.89, *r* = 0.01]. In short, there is no difference between conditions in terms of absolute attitude change. Post-hoc analyses using attitude change divided by the attitude at pre-test as an outcome revealed similar results.

The same goes for self-reference and psychological distance: There is no interaction between the factors self-image and behavioral focus [*F*_(4, 456)_ = 0.85, *p* = 0.50, $\eta_{p}^{2}$ = 0.007; *F*_(4, 456)_ = 0.86, *p* = 0.49, $\eta_{p}^{2}$ = 0.007 for self-reference and psychological distance respectively], nor a main effect of self-image [*F*_(2, 460)_ = 1.27, *p* = 0.28, $\eta_{p}^{2}$ = 0.005; *F*_(2, 460)_ = 1.47, *p* = 0.23, $\eta_{p}^{2}$ = 0.006] or behavioral focus [*F*_(2, 460)_ = 0.51, *p* = 0.60, $\eta_{p}^{2}$ = 0.002; *F*_(2, 460)_ = 0.13, *p* = 0.88, $\eta_{p}^{2}$ = 0.001]. The contrasts revealed no difference either between (1) the optimal combination of parameters for use for self-reevaluation vs. the control group [*t*_(456)_ = 0.59, *p* = 0.55, *r* = 0.03; *t*_(456)_ = 0.14, *p* = 0.89, *r* = 0.01]; (2) for anticipated regret vs. the control group [*t*_(456)_ = −0.36, *p* = 0.72, *r* = 0.02; *t*_(456)_ = −0.04, *p* = 0.97, *r* < 0.01]; and (3) for self-reevaluation vs. anticipated regret [*t*_(456)_ = 0.94, *p* = 0.35, *r* = 0.04; *t*_(456)_ = 0.10, *p* = 0.92, *r* < 0.01], nor for the comparison between the control group and all other conditions [*t*_(456)_ = −0.41, *p* = 0.69, *r* = 0.02; *t*_(456)_ = −0.81, *p* = 0.42, *r* = 0.04].

#### Secondary outcome

A total of 123 participants (27.0%) were ambivalent with regard to their attitude toward OCP at pre-test and 129 participants (28.1%) were ambivalent at post-test. When looking at the change between pre- and post-test measurement, 27 participants became less ambivalent (6.0%), 390 participants did not change (86.9%), and 31 participants became more ambivalent (7.1%). There is no interaction between the factors self-image and behavioral focus [$\chi_{\left(8,\;\text{N}\;=\;466\right)}^{2}$ = 11.28, *p* = 0.19], nor a main effect of self-image [$\chi_{\left(4,\;\text{N}\;=\;466\right)}^{2}$ = 4.01, *p* = 0.41] or behavioral focus [$\chi_{\left(4,\;\text{N}\;=\;466\right)}^{2}$ = 4.13, *p* = 0.39].

## Discussion

The key finding of the current study is that the behavior change methods of self-reevaluation and anticipated regret did not have an impact on attitude change in an online context, nor on the distance perceived by participants. Therefore, the focus of this section is on explaining these findings in the light of the characteristics of both the study design and the sample. In terms of manipulation, affective assessment of self-image resulted in higher scores on stimulating self-reevaluation, but this was not the case for cognitive assessment of self-image. Hence, it could be that the manipulation partly failed. Although the expression of “thoughts” was stressed in the manipulation by using italics and bold, it might be that participants interpreted thoughts as referring to both cognitive and affective assessment. Examination of the answers provided by participants, however, did not confirm this possible explanation. Moreover, affective assessment of self-image resulted in higher scores on stimulating self-reevaluation, making it an adequate manipulation of anticipated regret.

The lack of impact on attitude change could be partly attributed to the subtleness of the manipulation, which can be deemed a low intensity intervention. Originally, self-reevaluation has been applied by means of interaction between patients and health professionals such as psychotherapists (Medeiros and Prochaska, 1988) or specialist nurses (Van Kesteren et al., 2006). The intensity of such interventions can be deemed higher and this also provides the possibility to guide participants in the process of self-reevaluation. However, comparable to the low intensity in the study at hand, paper-based delivery of manipulations that participants have to complete by themselves were successful for both self-reevaluation (Armitage, 2009) and anticipated regret (Richard et al., 1996; Hetts et al., 2000). With regard to anticipated regret, even just letting participants complete one close-ended question whether they would regret it if they did not perform a certain behavior, without letting them write down their thoughts, has been shown to be a successful manipulation (Sheeran and Orbell, 1999). In short, even subtle manipulations such as the ones in the study at hand can be successful. So, the null results cannot be fully contributed to the subtleness of the manipulation. Furthermore, it needs to be stressed that the attitude at pre-test was already quite positive (*M* = 5.48), although this still leaves room for improvement on a scale ranging from 1 to 7. Also, even though the information text consisted of objective medical evidence, the fact that participations had to read the information text before the actual manipulation might have affected the self-reevaluation.

Another more likely explanation for the lack of impact is the use of the Internet as delivery channel, because previous studies regarding self-reevaluation and anticipated regret were not conducted in an online context. In the field of survey methodology, the comparison between paper-based vs. Internet-based questionnaires has received a lot of attention. For example, previous studies found that data quality of Internet-delivered questionnaires is not adversely affected by non-serious responders (Gosling et al., 2004) or social desirability (Crutzen and Göritz, 2010, 2011). This topic has received less attention in the area of Internet-delivered interventions aimed at promoting healthy behavior, although there is some evidence that printed intervention materials are better used in comparison with Internet-delivered intervention materials (Peels et al., 2013). Furthermore, in the online context of this study, there was no experimenter present. Previous work demonstrated that the mere physical presence of an experimenter had a positive effect on, for example, participants completing a simple word construction task (Rittle and Bernard, 1977). It might be that the online context differs from paper-based delivery of comparable manipulations, because of characteristics of the delivery channel and the lack of presence of an experimenter. Future research is needed to shed more light on differences between delivery channels, by comparing these manipulations across different delivery channels and with and without the presence of an experimenter.

The focus on young women's attitudes toward OCP was chosen based on the assumption that there would be a certain amount of attitudinal ambivalence and, hence, their attitudes would be more malleable (Petty and Cacioppo, 1986). Although ambivalent attitudes are weak predictors of behavior, they have high impact on information processing (Dalege et al., 2016) and, therefore, are more pliable to change (Armitage and Conner, 2000). However, only 27.0% of the participants in our study were ambivalent with regard to their attitude toward OCP at pre-test. A possible explanation might be that the young women who participated in our study had reached the age of majority (*M* = 21.3 years), while the decision to start using OCP is often made earlier in life (Thorogood and Vessey, 1990). Hence, it might be that based on their previous experience, participants in our study already hold strong attitudes toward OCP (either in favor or against). Stability over time is one of the defining attributes of strong attitudes (Krosnick and Petty, 1995). Previous research regarding the role of anticipated regret also showed that intention was a better predictor of behavior at high levels of stability, compared to moderate and low levels of stability of intention (Abraham and Sheeran, 2003). It needs to be stressed, however, that OCP use was not assessed, which is a limitation of this study.

There was no change in attitudinal ambivalence for the majority of participants (i.e., 86.9%). It is still possible, however, that participants' attitudes were linked to evaluative associations of opposite valence (Petty et al., 2007). In the study at hand, there was no desired direction in terms of stimulating participants to be in favor or against OCP. Using or not using OCP was not being valued as “good” or “bad” behavior. This is in contrast with most Internet-delivered interventions, in which the desired direction is often made explicit (e.g., stimulating participants to be more physically active or to abstain from smoking) (Kohl et al., 2013). It could be that the lack of desired direction resulted in participants merely affirming their existing evaluative associations, thereby decreasing the likelihood of detecting changes in attitude or attitudinal ambivalence in this study. A previous study demonstrated that if information provided is in contrast with existing evaluative associations, then people engage in greater information processing as if they are attempting to resolve this ambivalence (Petty et al., 2006).

Despite the null results, the current study contributes to the body of evidence regarding self-reevaluation and anticipated regret. Of course, one study is no study (Lakens et al., 2012), but the use of a factorial design enables us to gain more insight into possible interactions between parameters for use of both behavior change methods (even when they are not effective). This type of studies is needed to acquire a robust evidence base for our toolbox of behavior change methods. Future studies are needed to replicate the findings of this specific study (Lindsay, 2015). Subsequently, this evidence can be integrated in meta-regressions of experimental studies to advance behavior change theory (Peters et al., 2015).

## Ethics statement

This study was carried out in accordance with the recommendations of Simon Fraser University Research Ethics Board (file number: 2015s037) as well as the Ethical Committee Psychology of Maastricht University (ECP-149) with online informed consent from all subjects. All subjects gave online informed consent in accordance with the Declaration of Helsinki. The protocol was approved by the the Simon Fraser University Research Ethics Board as well as the Ethical Committee Psychology of Maastricht University.

## Author contributions

Study conception and design: RC, DC, ST, EL, and RR; acquisition of data: RC, ST; analysis of data: RC, ST; interpretation of data: RC, DC, ST, EL, and RR; drafting of manuscript: RC, DC, ST, EL, and RR. All authors agree on the final version of the manuscript.

## Funding

This study was supported by an Insight grant from the Social Sciences and Humanities Research Council of Canada (SSHRC). The funder had no role in study design, data collection and analysis, decision to publish, or preparation of the manuscript.

### Conflict of interest statement

The authors declare that the research was conducted in the absence of any commercial or financial relationships that could be construed as a potential conflict of interest.
